# Cancer risks from cosmic radiation exposure in flight: A review

**DOI:** 10.3389/fpubh.2022.947068

**Published:** 2022-11-22

**Authors:** Christopher Scheibler, Sneh M. Toprani, Irina Mordukhovich, Matthew Schaefer, Steven Staffa, Zachary D. Nagel, Eileen McNeely

**Affiliations:** ^1^Environmental and Occupational Medicine and Epidemiology Program, Harvard T.H. Chan School of Public Health, Boston, MA, United States; ^2^John B. Little Center for Radiation Sciences, Department of Environmental Health, Harvard T.H. Chan School of Public Health, Boston, MA, United States; ^3^Department of Environmental Health, Harvard T.H. Chan School of Public Health, Boston, MA, United States; ^4^Department of Surgery, Boston Children's Hospital, Harvard Medical School, Boston, MA, United States

**Keywords:** flight attendant, pilot, aircrew, cosmic ionizing radiation, cancer, aerospace, military

## Abstract

Aircrew (consisting of flight attendants, pilots, or flight engineers/navigators) are exposed to cosmic ionizing radiation (CIR) at flight altitude, which originates from solar activity and galactic sources. These exposures accumulate over time and are considerably higher for aircrew compared to the general population, and even higher compared to U.S. radiation workers. Many epidemiological studies on aircrew have observed higher rates of specific cancers compared to the general population. Despite high levels of CIR exposure and elevated rates of cancer in aircrew, a causal link between CIR and cancer has yet to be established. Many challenges still exist in effectively studying this relationship, not the least of which is evaluating CIR exposure separately from the constellation of factors that occur as part of the flight environment. This review concentrates on cancer incidence and mortality observed among aircrew in epidemiologic studies in relation to CIR exposure and limitation trends observed across the literature. The aim of this review is to provide an updated comprehensive summary of the literature that will support future research by identifying epidemiological challenges and highlighting existing increased cancer concerns in an occupation where CIR exposure is anticipated to increase in the future.

## Introduction

Flight attendants (FA) and pilots are consistently exposed to a complex variety of physical, chemical, biological, and psychosocial stressors. Physical exposures during flight include cosmic ionizing radiation (CIR), decreased oxygen levels, high noise and vibration levels, radiofrequency radiation, electromagnetic fields, and potentially ultraviolet radiation (UV). Chemical exposures in the aircraft include jet fuel and engine oil combustion products, ozone, flame retardants, pesticides, and disinfectants (1). Infectious biological agents pose a significant concern with today's global travel as seen in the recent SARS-CoV-2 pandemic (2, 3). In addition to these exposures, aircrew perform physically and psychologically demanding work that includes circadian rhythm disruption due to shift work and crossing time zones as well as potentially stressful interaction with passengers (1, 4). Historically, FA have also been exposed to high levels of secondhand tobacco smoke in the aircraft cabin (5). The profiles of these exposures are unique during air travel, and therefore it is difficult to untangle a specific exposure from the overall “flight environment” to understand its respective health impacts.

Ionizing radiation (IR) is a known human carcinogen and a causal risk factor for non-melanoma skin cancer (NMSC) and cancers of the breast, salivary gland, esophagus, stomach, colon, lung, bone, kidney, urinary bladder, brain/central nervous system, and thyroid (6). Studies regarding melanoma and IR are equivocal, but positive associations have been observed in some studies (7). Association have also been observed with other cancers such as rectal, liver, pancreatic, ovarian, and prostate, but not at the level required to assess causality (6). As opposed to the IR literature that incorporates direct measures of exposure, most CIR studies in aircrew primarily use employment tenure as a proxy of CIR exposure in lieu of direct measures, and therefore a complex mix of all flight-related exposures is considered rather than radiation alone. The potential links between specific exposures such as CIR and pathological mechanisms remain unclear, and therefore data stratifying exposure relationships such as CIR dose-response investigations are valuable to increase the understanding of the health risk among aircrew. It is also important to note that cumulative CIR exposure levels observed in studies often exceed expert-informed guidelines created to protect workers and the public from possible radiation-induced health effects. Despite CIR being recognized as a hazard, there still exist no established official dose limits, dosimetry surveillance requirements, or associated mandatory training for U.S. aircrews. By contrast, the European Union (EU) requires airlines to assess exposure of aircrew when the effective dose to the crew is expected to be above 1 mSv/yr. In these situations, airlines consider the assessed exposure when organizing working schedules to maintain doses below 6 mSv/yr, and they are required to inform workers of the health risks associated with their work duties in addition to discussing their individual dose (8, 9).

CIR is comprised of galactic cosmic radiation (GCR) that originates from outer space created by distant explosive events such as supernovas, and solar cosmic radiation (SCR) that is created by solar activity and characterized by solar particle events (SPEs) (10). There are two important differences between SCR and GCR. First, SCR exposure is directionally oriented based on the positioning of the sun, in contrast to the omnidirectional nature of GCR. Second, SCR has more particles at lower energy as compared to GCR. There are also important interactions between GCR and SCR. The magnitude of GCR and SPEs both oscillate with the 11-year solar cycle because the strength of the sun's magnetic field affects the amount of GCR that reaches the earth. At the solar maximum, more frequent and intense SPEs are accompanied by decreased GCR reaching Earth. Conversely, during solar minimum, the decreased solar activity allows higher levels of GCR to reach our planet. Estimates of past worst case SPE exposure at cruising altitude during polar routing show increases in CIR magnitude up to 9000% as compared to levels experienced on the Earth's surface (11). As an example, since the start of SPE observation record-keeping in 1942 the most intense SPE took place on 23 February 1956 and based on worst case estimates the event would have theoretically caused subsonic (~35,000 feet) and higher altitude supersonic (~55,000 feet) aircrew and passenger CIR exposures of up to 4.5 and 6.1 mSv, respectively (11). This equates to roughly 23–31% of the International Commission for Radiation Protection (ICRP) annual occupational exposure limit and is well above the 1 mSv annual limit recommended for passengers, however it is important to note that historically the majority of recorded SPEs have not approached levels that would cause increased doses of >1 mSv (11). Historically, the prediction of significant SPEs that would allow for the warning of aircrew and passengers has not been possible, but current efforts are in development to validate SPE modeling. Human exposure occurs when energetic charged particles (protons and alpha particles) from CIR sources outside of the solar system collide with elements in the earth's atmosphere (such as nitrogen and oxygen) to create a cascade of sub-atomic particles, or when charged particles are released during solar flares (10). During exposure the composition of CIR is predominantly neutron particles, and overall is comprised of both low and high linear energy transfer (LET) radiation. The Earth's atmosphere and magnetic field shield against CIR, but this protection decreases with higher altitudes and more polar latitudes, thereby significantly impacting circumpolar flights operating at cruising altitudes of 35,000 feet or above (10). Overall, GCR levels are estimated to double for each 4,500 feet increase in altitude, and radiation levels at polar latitudes are approximately twice as high as at the equator (10). Although SPE exposure during flight is rare, SPEs have the potential to expose aircrew to higher levels of CIR during a single event than might be encountered in over a year of flight related GCR exposure (11). At flight altitude, in addition to GCR and SPEs, aircrew are potentially subjected to a variety of other radiation exposures to include solar neutron events and solar gamma-ray events, as well as terrestrial gamma-ray flashes that are associated with thunderstorms and lightning (12). The impact of these additional radiation sources is yet to be fully understood or accounted for in exposure estimations (13, 14). Aircrew may also be exposed to other non-CIR radiation sources such as radioactive cargo, airport security scanners and medical imaging related to occupational medical surveillance requirements, however there is a lack of literature evidence evaluating the magnitude of these exposures.

There exist many challenges in assessing CIR exposure and understanding the impact on human health. Comparison of flight-related CIR exposure to other human exposures to IR is challenging because studied health effect outcomes have been based on single events such as nuclear disasters or atomic bomb detonation with extremely high dose acute exposures, which differ with respect to particle compositions as well as dose magnitude and period of exposure. Relating risk associated with flight-related CIR exposure to naturally occurring background sources of radiation is also difficult. Directly measuring CIR levels at altitude can be difficult. Measurement devices have historically been cumbersome and unreliable, and other than in the case of the Concorde, aircraft manufacturers have not prioritized the placement of permanent radiation dosimeters into aircraft design. Without onboard equipment, experimental measurements have been performed with temporary dosimeters that are unable to measure aircrew cumulative dose measurements over longer time periods. The International Organization for Standardization (IOS) defines how instruments for measuring CIR should be calibrated and how dosimetry should be performed to standardize results across different assessments (15). There are several private and publicly available models for estimating CIR exposure at altitude. Commonly used models in the literature include AVIDOS, EPCARD, JISCARD EX, PANDOCA, FREE, PCAIRE, SIEVERT, and CARI, and additional information can be found in the European Radiation Dosimetry Group (EURADOS) report on CIR models (15). It is important to note that a EURADOS report evaluating models used to assess aircrew CIR exposure found strong agreement among the models, and recommended validation of all models with measured data meet an agreement threshold within ± 30% at a 95% confidence level (15). The models generally do not account for the SPE component of CIR, but work has been done to incorporate SPEs that have sufficient energy to be detected with neutron monitors on the earth's surface, which are termed ground level enhancement (GLE) events. By comparing historical GLE data to previous CIR dosimetry taken on-board Concorde aircraft during the same SPE, SiGLE modeling has been created to provide estimates of SPE radiation received at altitude using GLE data, and this modeling has been incorporated into the SIEVERT model (11, 15). Some recent direct measurements of CIR onboard aircraft exist, and they are important for validating computer codes by evaluating the radiation dose on board aircraft e.g., intercomparison campaigns REFLECT (16) and CONCORD (17). However, the majority of models do not incorporate SiGLE modeling, and that this SPE estimation does not account for SPEs that do not reach the threshold to be recorded as GLE events, which could still be significant exposures at altitude. Given the variability of potential CIR exposure due to the solar cycle, effects of altitude and latitude, and random impacts of SPEs, there is a need for accurate individualized dosimetry to understand CIR-related health risk in aircrew. Software models have been created from direct measurements that estimate CIR exposure based on specific flight characteristics (origin, destination, route, date, etc.), though these platforms also vary in their estimations (15), and these approaches generally do not account for SPEs. To mitigate these challenges, investigators have developed a variety of approaches for assessing CIR exposure in aircrew. Generally, two main types of investigations are reported in the literature: measurement studies using dosimeters/modeling to characterize CIR exposure at altitude or cohort studies seeking to characterize health effects associated with CIR exposure generally based on aircrew employment metrics without associated dosimetry. Measurement studies utilize personal dosimeters and instruments onboard aircraft or employ exposure estimation modeling based on subject and flight characteristics. Cohort studies use a variety of surrogates for CIR exposure proxy including employment as aircrew, duration of employment, subjective self-report or employer records of flight hours/frequency/route and exposure matrices, in addition to applying validated CIR modeling. The literature is dominated by retrospective cohort studies that can utilize available cancer incidence and mortality data, however, usually limits the ability to conduct a detailed exposure history. For this reason, most studies have crude estimates of subject flight exposure that are limited to employment alone or duration of employment as aircrew, and studies linking detailed subject exposure data with health outcomes are scarce.

In summary, although the literature regarding aircrew health risk demonstrates evidence of increased rates of some cancer outcomes, a causal relationship has yet to be established. Furthermore, aircrew are not classified as radiation workers in many countries including the U.S, and their CIR exposures are therefore often not regulated. In support of these challenges, this comprehensive literature review focuses on CIR exposure and cancer risk observed in aircrew. Synthesis of this knowledge is important to inform clinical and occupational health guidelines as well as to identify future research priorities that would further assist in protecting the health of aircrew. An accurate understanding of occupational exposure is also important in the context of public health recommendations for safeguarding aerospace travelers, especially frequent flyers whose CIR exposure may approach that of aircrew.

## Methodology

This review is based on a comprehensive analysis of all epidemiological CIR-related literature published through 2021 evaluating health outcomes in aircrew. The literature demonstrates a wide variety of health outcomes to consider, with most of the studies evaluating cancer incidence and mortality risk. Based on the evidence of association from previous studies, the total number of investigations of different outcomes available in the literature, and outcome causal associations with IR and theorized relationships with CIR exposure, this review primarily focuses on melanoma skin cancer, non-melanoma skin cancer, breast cancer, prostate cancer, lymphoma, leukemia, thyroid cancer, and brain/CNS cancer. Although the review focuses on these specific cancer outcomes, other cancer diagnoses of investigation in the literature are discussed more broadly. Studies investigating all-cancer outcomes generally document lower rates of all-cancer incidence and mortality as compared to the general population (18–23). Lower rates in aircrew could reflect occupational screening factors as previously discussed, and as these outcomes are regularly corroborated across studies, this review will not discuss specific study results for all-cancer rates that are found in the literature.

### Cancer risk and mortality

Each of the following sections discuss forest plot figures (made using Prism 8.1 software) summarizing the effect estimates in the literature for each cancer diagnosis. These figures contain estimates for both FAs (termed “cabin aircrew”—CAC) displayed in blue and pilots (termed “cockpit aircrew”—COC) displayed in red. In cancer outcomes summarized for both sexes, male estimates are displayed on the left and female estimates are displayed on the right. In each figure, incidence studies are grouped above mortality studies and separated by a dashed dividing line, and within the incidence/mortality sections the FA studies are grouped above pilot studies. For cancer diagnoses that have sub-diagnoses (e.g., Lymphoma: All Lymphoma, Non-Hodgkins, Hodgkins), estimates are also grouped by sub-diagnoses and signified by specific estimate shapes (circle, square, diamond, triangle) explained in the legend. Study author, year and type of effect estimate are listed on the y-axis, and meta-analysis studies are signified with the letter “M”. The studies are ordered based on ascending aircrew sample size used in the study (largest samples will be above smaller samples), while adhering to the incidence/mortality, FAs/pilots, and sub-diagnosis groupings. Finally, due to the forest plot figure design scheme being separated by sex, the small number of studies that only reported a combined male/female effect estimate are not represented in the figures but are discussed in the respective text section. Additionally, a table summarizing all the commercial aircrew studies referenced in forest plot figures has been provided (Table 1).

**Table 1:** Overview of CIR related cancer studies and cohorts.

**References**	**COC M, F**	**CAC M, F**	**Estimate**
**United Kingdom**			
Irvine and Davies (24)	NR, 0		M
Irvine and Davies (25)^**A**^	7362, 0		M
De Stavola et al. (26)^**B**^	15881, 446		M
dos Santos Silva et al. (27)	15867, 462		I
**Finland**			
Pukkala et al. (28)^**C**^		187, 1577	I
**Denmark**			
Lynge (29)		0, 915	I
Gundestrup and Storm (30)^**D**^	3790, 87		I
**Norway**			
Haldorsen et al. (31)^**E**^	3701, 0		I
Haldorsen et al. (32)^**F**^		588, 3105	I
**Iceland**			
Rafnsson et al. (33)	458, 0		I
Rafnsson et al. (34)^**H**^		158, 1532	I
Gudmundsdottir et al. (35)	551, 0		I
**Sweden**			
Hammar et al. (36)^**I**^	1490, 0		I
Linnersjö et al. (37)^**J**^		632, 2324	I
**Italy**			
Ballard et al. (38)^**K**^	3022, 0	3418,3428	M
**Greece**			
Paridou et al. (39)^**L**^	843, 0	1835 (M+F)	M
**Canada**			
Band et al. (40)	913, 0		I, M
Salisbury et al. (41)	NR, 0		M
Band et al. (18)	2680, 0		I, M
**Australia**			
Olsen et al. (42)	NR, 0		I
**United States**			
Wartenberg and Stapleton (43)		0, 287	I
Nicholas et al. (44)	NR, 0		M
Reynolds et al. (45)		1216, 6895	I
Pinkerton et al. (21)		1701, 9610	M
Pinkerton et al. (46)		0, 6095	I
Yong et al. (23)	5958, 6		M
Schubauer-Berigan et al. (47)		0, 6093	I
McNeely et al. (1)		998, 4368	I
**Large German Cohort**			
Zeeb et al. (48)^**N**^	6061, 0		M
Blettner et al. (49)^**O**^		4537, 16014	M
Zeeb et al. (20)	6017, 0	3735, 17022	M
Hammer et al. (50)	6006, 0		M
Dreger et al. (51)	6006, 90	3733, 17017	M
**Large Nordic CAC Cohort**			
Component studies:^C, F, H, J^			
Pooled analysis reports			
Pukkala et al. (52)		1559, 8507	I
**Large Nordic COC Cohort**			
Component studies: ^D, E, G, I^			
Pooled analysis reports			
Pukkala et al. (53)	10032, 0		I
Pukkala et al. (54)	10051, 0		I
**ESCAPE Cohort**			
Component studies: ^D, E, G, I, K, N^			
Pooled analysis reports			
Blettner et al. (55)^**A, L**^	27797, 0		M
Zeeb et al. (19)^**L, O**^		11079, 33063	M
Langner et al. (56)	19184, 0		M
Hammer et al. (22)^**A, B, L, M, O**^	36816, 0	12288, 44667	M

*COC, Cockpit Crew; CAC, Cabin Crew; NR, Not Reported; I, Incidence; M, Mortality; M, Male; F, Female.

It is important to note that aircrew data used is sometimes duplicated in reporting by aircrew cohort studies in the literature and that data sources may also be redundant in comparison of systematic reviews and meta-analyses. Due to their frequent reference, often overlapping sample use, and large study size, a brief overview of four major cohorts in the literature that are commonly referenced for pooled analysis evaluations is warranted.

The first is a large Nordic cohort that reports on cancer incidence in male and female FAs (52) and is comprised of cohorts from four individual studies (28, 32, 34, 37).

The second large cohort is also from the Nordic region but reports on cancer incidence in pilots from five countries (53, 54) and is comprised of cohorts from four individual studies (30, 31, 33, 36).

The third large cohort is the European Study of Cancer Risks Among Airline Personnel (ESCAPE) cohort that was established in 1997 and includes cohorts from previous studies of aircrew from 9 European countries (25, 30–33, 36, 38, 39, 48). The ESCAPE cohort incorporates all the individual studies from the large Nordic male pilot cohort. The initial two investigations of the ESCAPE cohort reported on male pilot mortality (55, 56), which was followed by a third study (19) that evaluated FA mortality and incorporated an additional cohort of German aircrew into the analysis (49). The most recent report from the ESCAPE cohort (22) evaluated mortality in both pilots and FAs utilizing the expanded cohort described by Zeeb et al. (19), but also added a UK cohort (26) and a U.S. cohort (21) into the analysis.

The fourth frequently used large cohort is comprised of German aircrew and investigations were first published in 2002 by two separate reports evaluating mortality risk in pilots (48) and FAs (49). Of note, the FA cohort described by Blettner et al., is the same cohort that was added in the third analysis of the ESCAPE cohort by Zeeb et al. discussed previously. The third report of the German aircrew cohort was a combined study of pilot and FA mortality followed by a fourth analysis that evaluated mortality only in pilots (50). The fifth and most recent follow-up of the large German cohort by Dreger et al. (51) again evaluates mortality in both pilots and FAs.

Certainly, there are differences in cohort specifics, study methods, CIR estimation techniques, outcomes of interests and follow-up periods across the different reports of the two Nordic cohorts, ESCAPE and German aircrew cohorts, however describing these details in totality is not feasible within this review and distinctions for specific studies are discussed within each outcome section. Overall, it is generally assumed that the effect estimates reported in the most recent follow-up of each of the four cohorts (22, 51, 52, 54) to be more accurate given evolving CIR estimation techniques, increasing surveillance periods and larger sample sizes, however this assumption has not been validated.

## Results

### Breast cancer (BC)

**Figure 1:**
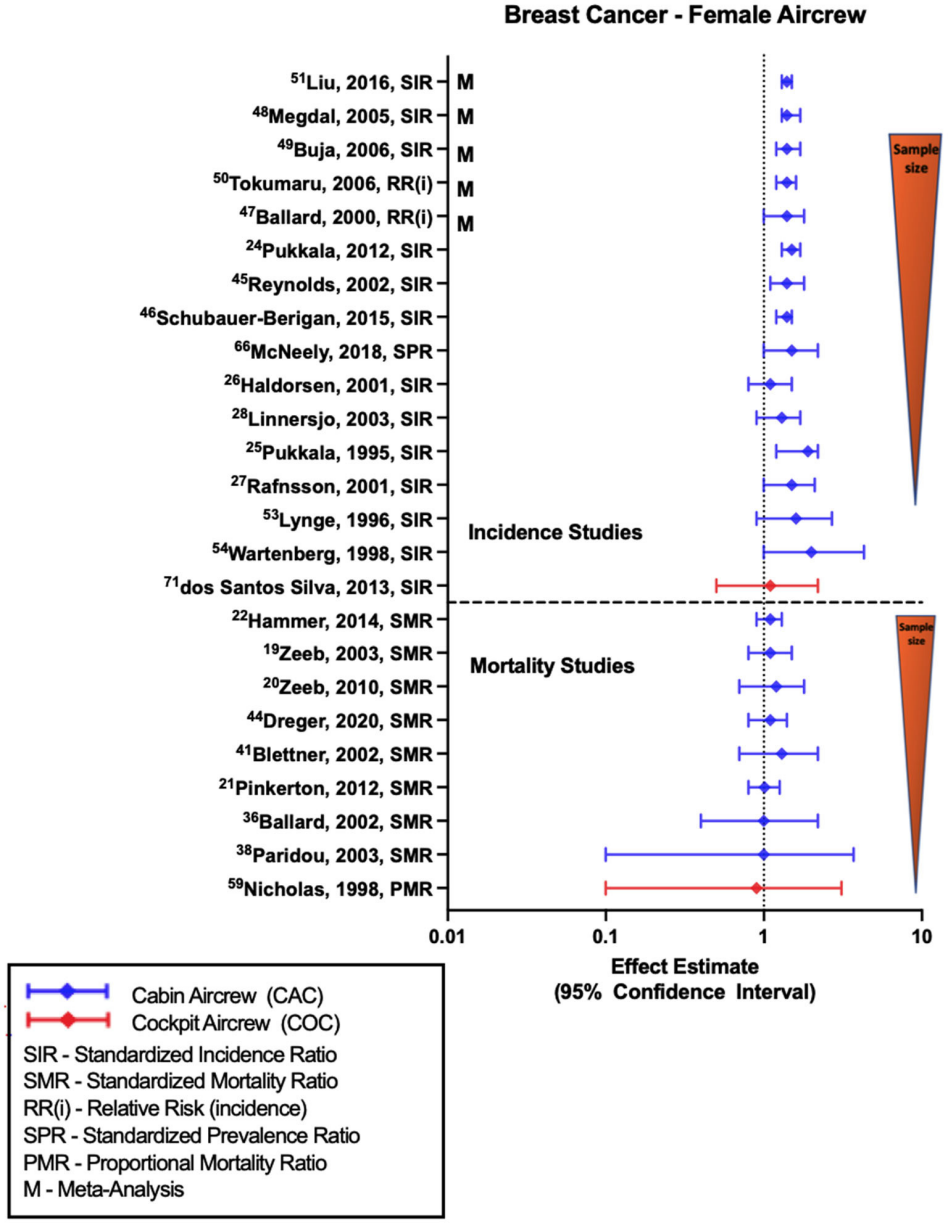
Forest plot for studies evaluating breast cancer incidence or mortality ratios among female aircrew in association to CIR exposure.

Most retrospective cohort studies evaluating BC incidence or prevalence among female FAs have shown associations with employment as cabin crew (Figure 1), as demonstrated in the reported standardized incidence ratio (SIR) of 1.50 (95% CI: 1.32, 1.69) from the large Nordic cohort (52) that was consistent with three large U.S. FA studies (1, 45, 47) as well as meta-analyses and systematic reviews (57–61). In contrast, differing estimates of equivocal findings with work as a FA in general (32) or specifically after 1971 (62) have been observed. In the Rafnsson et al. study, 1971 represented the year wherein the commercial jet aircraft was introduced in Iceland and was related to a presumed increase in CIR exposure from work as aircrew after 1971 related to exposure to higher flight altitudes, however an association was only seen in those working as a FA prior to 1971.

Smaller retrospective cohort studies have also yielded similar findings of associations or near-significant positive estimates with work as a female FA (28, 29, 34, 37, 43). CIR dose-response analyses have shown some evidence of a relationship with BC risk based on employment duration or estimated CIR exposure dose (28, 34, 45, 47, 62), however a similar number of investigations found no observable relationship with these metrics (32, 37, 52, 63) and in analyses with significant findings, there are still challenges with demonstrating trends across all exposure groups. When specifically stratifying by higher parity FAs, BC risk was found to be associated with cumulative CIR exposure based on employment duration (1) and CIR dose estimates (47, 64), although these studies also demonstrated a dose-response relationship between BC and circadian rhythm disruption (47, 64). This finding is especially interesting given the fact that in general BC risk is lower in multiparous women as compared to nulliparous, which increases suspicion for CIR as a causal agent, however an alternative theory is that the combination of a sleep-stressed home (as seen with higher parity) and an occupation with significant sleep-impacting shift work could synergistically lead to an increased risk of BC (47). Evaluation using lag time from the start of employment as a FA and BC risk did find evidence of a stronger association after a lag time of 20 years (34), which is consistent with what is known about non-hematologic cancer induction and latency periods (65). The only study to evaluate BC risk in female cockpit crew (with cohort size of 462 females), as opposed to FAs, observed a non-significant positive estimate with employment as aircrew (26).

Studies of BC mortality (Figure 1) consistently show equivocal findings or non-significant positive estimates based on employment as a FA demonstrated in observations from the ESCAPE cohort and associated component studies (19, 21, 22, 38, 39), and from follow-ups of the large German cohort (20, 49, 51). A recently published pooled analysis of BC in female FAs reported an SPR of 1.08 (95% CI: 0.37, 1.59) and SMR of 1.8 (95% CI: 0.63, 4.25), however these estimates were not included in (Figure 1) due to questions concerning the cited cohort studies included in the analysis with potential for duplication/error (66). The only study to cite female cockpit crew BC mortality observed a null finding based on using a crude proportional mortality ratio investigation (44). CIR dose-response analysis based on cumulative dose estimates (21, 51), employment duration (19, 20) and circadian rhythm disruption (21) showed no evidence of relationship with BC mortality across exposure categories, even with application of 10- and 20-year lags. Specific limitations in BC investigations include lack of control for confounders and challenges in comparison to the general population as BC risk has been shown to be variably modified by specific lifestyle and reproductive factors, and therefore it is difficult to assess FA risk as compared to the general population given observed differences in reproductive history and medication use, alcohol/tobacco use, exercise, and social habits between the groups (67).

### Melanoma skin cancer (MSC)

**Figure 2:**
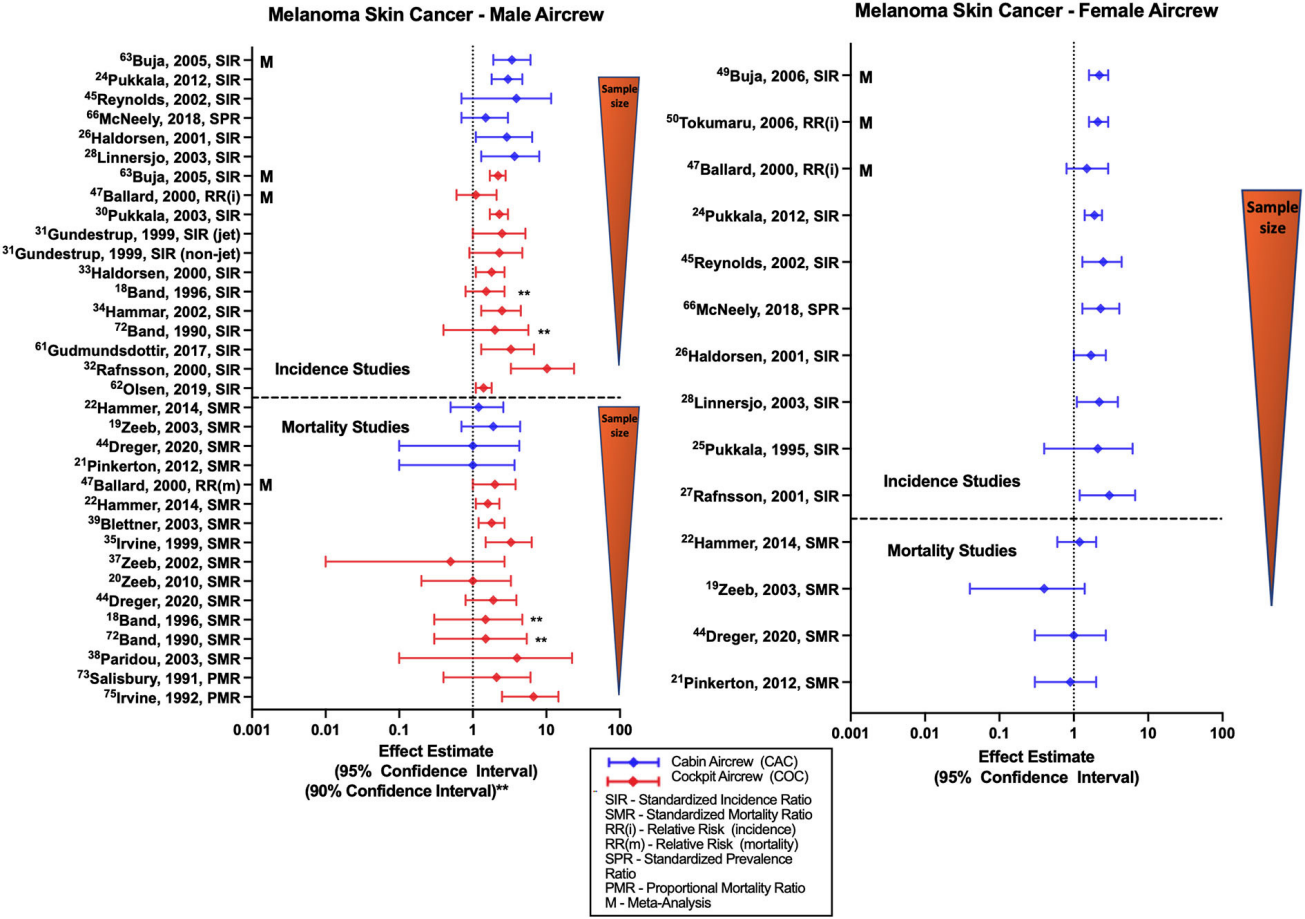
Forest plot for studies evaluating melanoma skin cancer incidence or mortality ratios among male **(left hand side graph)** and female aircrew **(right hand side graph)** in association to CIR exposure.

Studies of MSC incidence generally report associations with employment as aircrew (Figure 2) as demonstrated by female and male FA SIRs of 1.85 (95% CI: 1.41, 2.38) and 3.00 (95% CI: 1.78, 4.74) reported in the large Nordic cohort and supported by incorporated component studies (32, 34, 37). The large Nordic pilot cohort also found increased risk of MSC associated with work as male cockpit crew (SIR 2.29, 95% CI: 1.73, 2.98) (54), consistent with associations observed in smaller cohort studies (31, 33, 35, 36). This association was also seen in the single study of a Southern Hemisphere pilots (42). Studies show consistent findings by sex and aircrew position, summarized in literature meta-analyses (57–60, 68–70). CIR dose-response investigations based on employment duration or cumulative CIR dose estimates in Nordic male pilot cohorts report evidence of associations between MSC and higher doses or years of employment. CIR dose-response analysis in FAs have observed similar associations and non-significant positive trends however this risk relationship is not observed across all studies evaluating CIR exposure metrics (45, 46, 52).

Research has previously reported significant correlation among the distinct long-term trends in the solar cycle and skin cancer risk (71), and studies of MSC risk among aircrew rarely adjust for recreational UV exposure or other melanoma risk factors such as hair color, eye color, skin type, sunscreen use, history of sunburn, or family history of skin cancer. However, it is reassuring that a study conducted among FAs and pilots from Iceland that observed an excess risk of melanoma in aircrew, did not report a substantial difference in these melanoma risk factors compared to the general population (72). A risk factor study using a cohort of Finnish female FAs also reported no considerable differences in skin cancer risk factor prevalence or risk scores as compared to the general population, and in a secondary nested case-control analysis evaluating a combined melanoma/basal cell cancer outcome observed an OR 1.43 (95% CI 1.01, 2.04) with increased host risk factors, however an assessment based on employment duration combined with average annual CIR doses found no increased risk (73). A study conducted by dos Santos Silva and colleagues compared MSC risk among cockpit aircrew, air traffic control officers (ATCO) that have similar night shiftwork circadian disruption, and the general U.K. population, finding increased incidence of MSC among aircrew and ATCOs relative to the general population, as well as an association between total flight hours and melanoma incidence (27). Further, the study reported the strongest risk factor for melanoma in aircrew and ATCOs was based on skin type defined as skin that burns easily when exposed to sunlight. These results may be indicative of a complicated association between work factors (including circadian rhythm disruption among aircrew and ATCOs), lifestyle, personal characteristics, and MSC risk among aircrew (27).

The relatively few studies of melanoma mortality conducted among FAs (Figure 2) consist mostly of the ESCAPE cohort and associated analyses (19, 21, 22), and the large German cohort (51), and overall do not report evidence of an association with employment as aircrew in either males or females. CIR dose-response investigations have also found no evidence of a significant relationship between melanoma mortality and employment duration, cumulative CIR exposure or circadian rhythm in FAs (21). In contrast, mortality studies among male pilots have found associations reported in ESCAPE cohort and associated studies (22, 25, 55), and non-significant positive estimates reported in smaller cohorts (18, 39–41). The large German cohort also observed a non-significant positive estimate that has emerged during the latest follow-up of the cohort (51). CIR dose-response investigations of male pilot MSC mortality in the ESCAPE and large German cohort failed to show clear evidence of an association between cumulative CIR dose and melanoma risk with only observations of non-significant positive estimates that did not exhibit a significant trend test across categories. Other CIR dose-response analyses based on employment duration and CIR estimations have also failed to demonstrate any evidence of significant relationships with MSC mortality in pilots (23). Meta-analyses and systematic reviews report elevated SMRs for MSC mortality among pilots, but not FAs, in comparison to the general population (57, 69, 70). A study by De Stavola et al. evaluating MSC mortality in pilots and ATCOs found a non-significant positive estimate of MSC mortality in a combined cohort of male and female pilots, and additionally observed a mortality rate in pilots that was twice that of ATCOs (26). Specific limitations in MSC investigations are the lack of adjustment for melanoma-specific risk factors including sun and indoor tanning exposure, history of sunburns, skin type, race/ethnicity, age, or family history.

### Non-melanoma skin cancer (NMSC)

**Figure 3:**
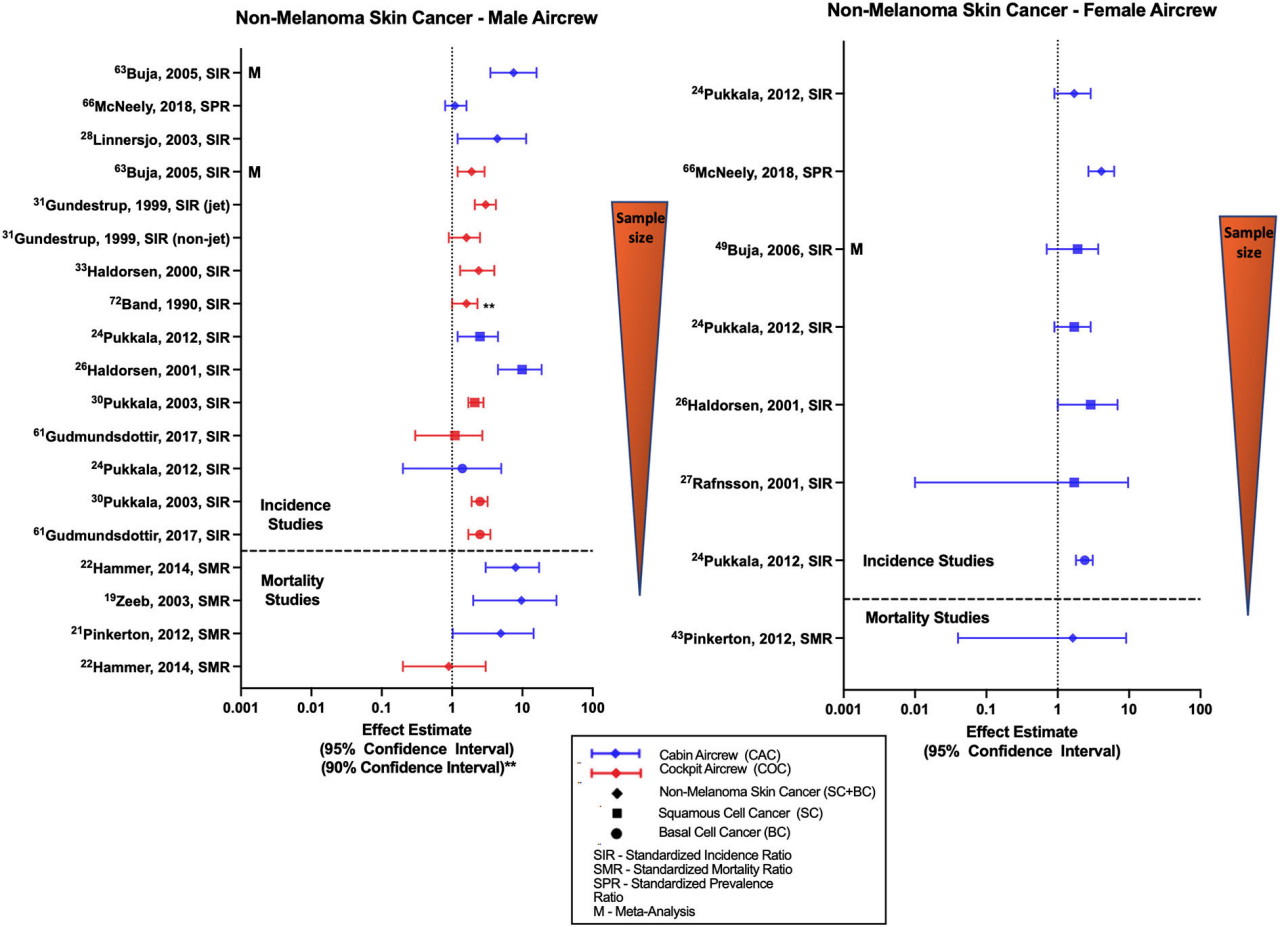
Forest plot for studies evaluating non-melanoma skin cancer incidence or mortality ratios among male **(left hand side graph)** and female aircrew **(right hand side graph)** in association to CIR exposure.

Most studies evaluating risk of all NMSC (basal cell and squamous cell carcinoma combined) with employment as aircrew have reported associations among male FAs, female FAs, and male pilots (Figure 3) (30, 31, 37, 40, 74). These associations are reflected in the findings of meta-analyses (68, 70) with a combined male/female pilot NMSC SIR of 1.86 (95% CI: 1.54, 2.25) (70). Studies evaluating outcomes of squamous cell and basal cell carcinoma individually (32, 34, 35), including the large Nordic FA and pilot cohorts and meta-analysis investigations also report similar associations and non-significant positive estimates in male and female aircrew. Studies that evaluated CIR dose-response relationships using cumulative CIR dose estimates or employment duration as aircrew have shown evidence with increasing exposure groups, observing both associations (30, 35, 54) and non-significant positive trends (31, 32, 74), however this finding was not observed across all investigations (52).

Likely due to the low associated mortality, few studies have evaluated NMSC mortality in aircrew and the literature mainly consists of observations from analyses of the ESCAPE cohort and associated component studies (19, 21, 22). Studies conducted among male FAs found associations between employment as aircrew and NMSC mortality with notably wide confidence intervals however the single study of mortality in female FAs did not observe a significant risk (21). Male pilot mortality evaluation in the ESCAPE cohort found an equivocal finding as compared to the general population (22), consistent with an observation from a combined male/female pilot cohort study (23). Overall, the increased risk of NMSC in aircrew demonstrated in the literature (Figure 3) is consistent with IR being a known causal risk factor for NMSC (6). Specific limitations in NMSC investigations are again the lack of adjustment for other skin cancer risk factors.

### Prostate cancer (PC)

**Figure 4:**
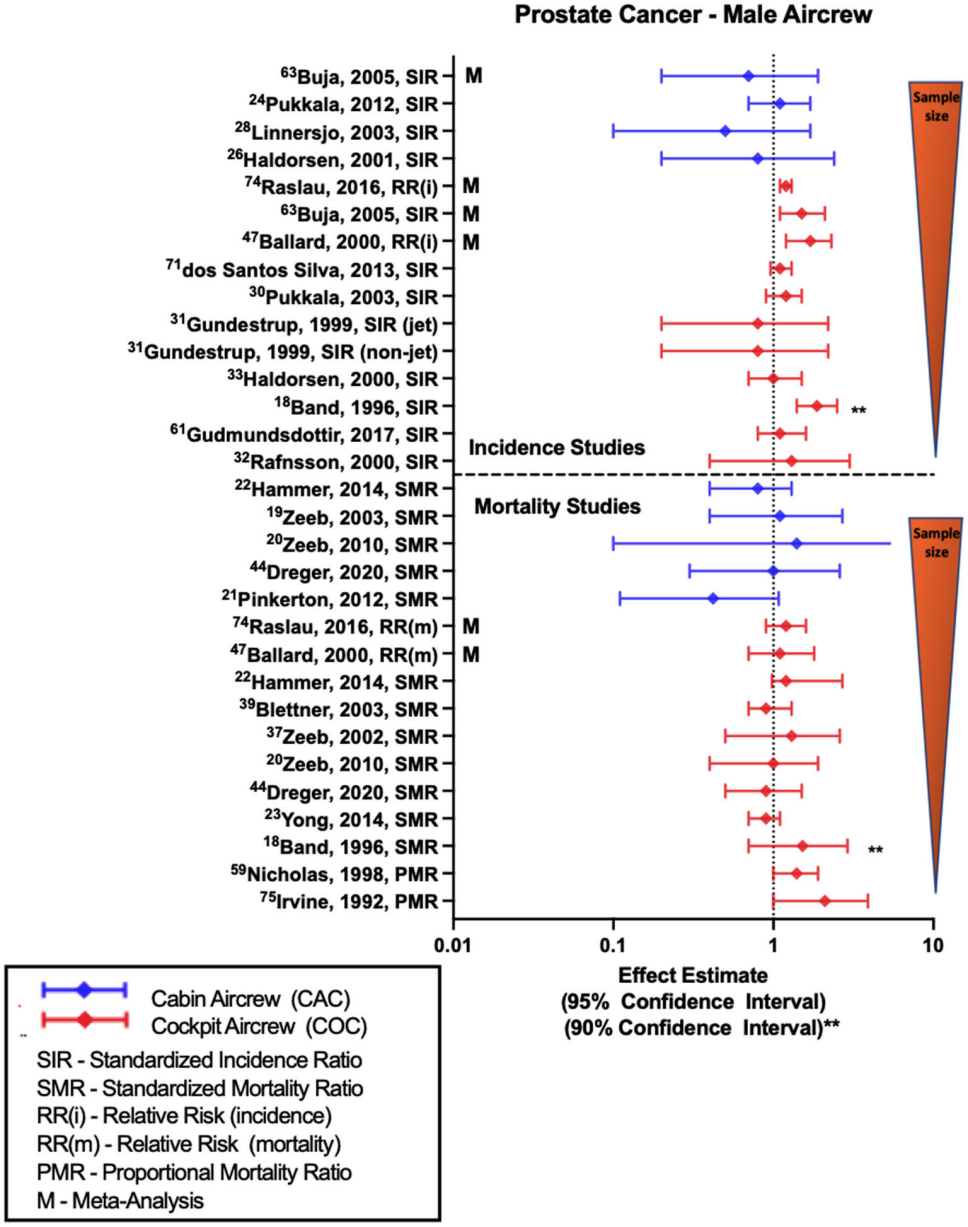
Forest plot for studies evaluating prostate cancer incidence or mortality ratios among male aircrew in association to CIR exposure.

Compared to BC, MSC, and NMSC, the risk estimates for PC among male FAs and pilots are less consistent (Figure 4). In pilot investigations based on employment as aircrew, non-significant positive estimates of prostate cancer were observed in the large Nordic pilot cohort (33, 54) and individual studies with one smaller study observing an association based on 90% confidence intervals (18). However, other investigations have also reported equivocal and one study did observe a non-significant negative risk estimate with PC (30). Multiple meta-analyses evaluating PC risk in pilots did report associations with employment as aircrew (57, 68, 75). In contrast to pilots, majority of studies evaluating PC risk in male FAs report either null findings as observed in the large Nordic cohort (52) or non-significant negative estimates as reported in Nordic component studies (32, 37) and meta-analysis (68). CIR dose-response investigations in male pilots did observe associations of significant risk with employment duration (35), career long-haul flight hours and annual CIR dose estimates (35, 54), however these trend findings were not consistent across all dose-response analyses (31).

Along the lines of incidence studies, few PC mortality studies have been conducted in FAs. Recent analysis of ESCAPE cohort and associated studies observe non-significant negative mortality estimates that differ from earlier analyses with no difference in the findings (19). In contrast, the most recent follow-up of the large German cohort observed an equivocal finding that differs from the non-significant positive estimate in an earlier analysis (20). Among pilot studies to include both ESCAPE and German cohorts, more investigations have observed null findings (20, 23, 51, 55), however two studies did report non-significant positive estimates including the most recent follow-up of the ESCAPE cohort (22, 48). Meta-analyses are mixed with non-significant findings reflecting both no relationship and positive mortality risk estimates (75). Studies with the largest non-significant positive estimates were also the smallest in terms of cohort size, and used 90% confidence intervals (18), as well as more crude proportional mortality study approaches (24, 44). Earlier CIR dose-response analyses of the large German pilot cohort (20, 48) did observe a non-significant positive trend in mortality risk with years of employment and CIR dose estimation suggesting a relationship, however these findings were not repeated in a subsequent follow-up employing an extended observation period (50).

### Lymphoma

**Figure 5:**
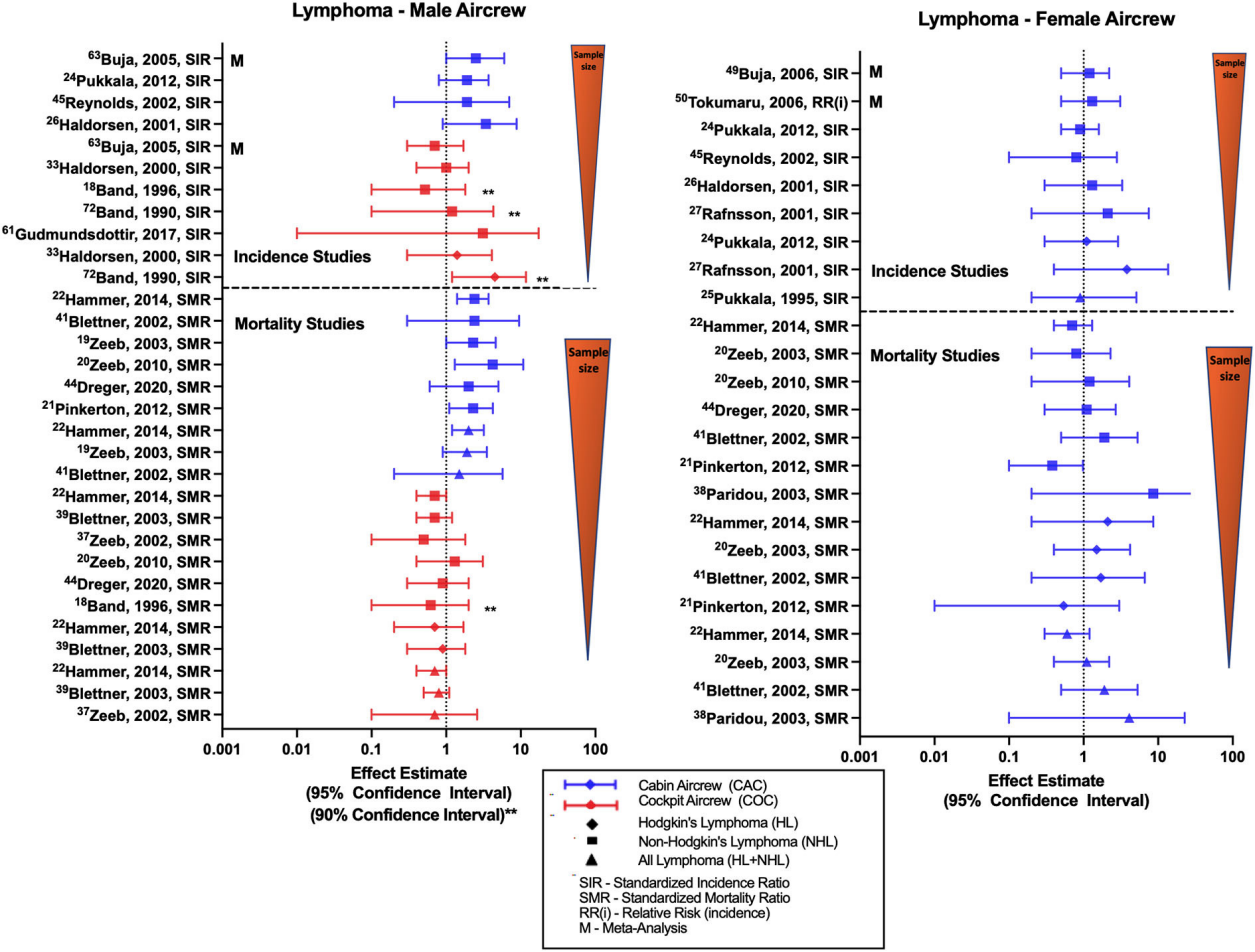
Forest plot for studies evaluating lymphoma incidence or mortality ratios among male **(left hand side graph)** and female aircrew **(right hand side graph)** in association to CIR exposure.

Studies evaluating lymphoma in aircrew generally report outcomes of all-lymphoma, non-Hodgkin's lymphoma (NHL), Hodgkin's lymphoma (HL) or a combination of these diagnoses. Overall female FA studies show mixed results, male aircrew observations differ by position with pilots and FAs having separate trends across studies (Figure 5). Male cabin crew investigations are limited to the outcome of NHL and observe non-significant positive estimates across cohort studies (32, 45, 52) and a significant meta-analysis SIR of 2.49 (95% CI: 1.03, 6.03) (68). The largest study evaluating NHL in male cockpit crew observed an equivocal finding that varied with both non-significant positive (35, 40), and negative estimates in smaller studies. Single meta-analysis of NHL incidence in male pilots reported a non-significant negative estimate (68). Studies of NHL in female cabin crew are also mixed and do not reach significance, observing both negative estimates in the large Nordic cohort (45, 52), and positive estimates (32, 34, 59, 60). The few studies evaluating HL risk generally observe positive estimates in male pilots (31, 40) and female FAs (34, 52).

Studies evaluating lymphoma mortality in aircrew show generally consistent but inverse findings in male cabin crew and male cockpit crew cohorts, and mixed results in female cabin crew (Figure 5). The most recent ESCAPE cohort analysis of NHL mortality risk in male FAs observed increased risk (SMR of 2.37; 95% CI: 1.41, 3.73) that was consistent with associations found in prior ESCAPE cohort analyses and component studies (21). These findings are positive but non-significant for mortality risk estimates reported in analyses of the large German cohort (20, 49, 51). As expected, higher NHL mortality rates also led to similar observations of higher all-lymphoma mortality rates in the same cohort studies of male FAs (19, 22, 49). When evaluating NHL in female cabin crew, the same ESCAPE cohort analyses and component studies found non-significant negative mortality estimates (19, 21, 22), however one component study observed a non-significant positive estimate (39). The most recent large German cohort follow-up observed a null NHL finding that differed with previous non-significant positive estimates in female FAs as compared to the general population (20, 39). Few HL mortality investigations in female FAs report non-significant positive estimates (19, 22, 49) except for one non-significant negative estimate report (21). As demonstrated in studies of male cabin crew, female all-lymphoma mortality findings vary based on the NHL and HL estimates observed in the respective studies (19, 22, 39, 49). Mortality studies of male pilots generally observe non-significant or near-significant negative risk estimates with NHL, HL and all lymphoma mortality as compared to the general population (18, 22, 48, 51, 55).

Overall, the lymphoma literature summarized in (Figure 5) generally shows non-significant positive incidence and significantly increased mortality NHL risk estimates in male cabin crew that was not observed repeated in female cabin or male cockpit crew. Additionally, studies evaluating combined male/female cockpit crew cohorts report non-significant negative incidence and mortality risk estimates (23, 27). There is some speculation that the consistently increased NHL mortality associations seen only in male cabin crew could potentially be associated with human immunodeficiency virus (HIV) and acquired immune deficiency syndrome (AIDS)-related disease, as studies have also demonstrated increased rates of Kaposi's sarcoma and HIV/AIDS mortality (19, 22, 46) in male FA cohorts. The potential outcome misclassification attributing HIV/AIDS deaths to NHL related to coding or recording errors with death certificate documentation and also prior to 1987, HIV related deaths were classified to deficiency of cell-mediated immunity, pneumocystis, malignant neoplasms including neoplasms of the lymphatic and hematopoietic tissues, and to a number of other causes (NCHS definitions, n.d.). Thus, some of deaths in the other causes category were also related to HIV-related disease leading to this misclassification which could lead to bias that would overestimate NHL mortality risk in male cabin crew. This misclassification hypothesis would also be consistent with findings that NHL mortality in male cabin crew was not found to be associated with exposure variables of employment duration or cumulative CIR dose (21). Other specific limitations in lymphoma investigations include lack of adjustment for risk factors such as age, overweight/obesity, family history, high risk chemical exposures (e.g., benzene, insecticides, etc.), autoimmune diseases and certain infections.

### Leukemia

**Figure 6:**
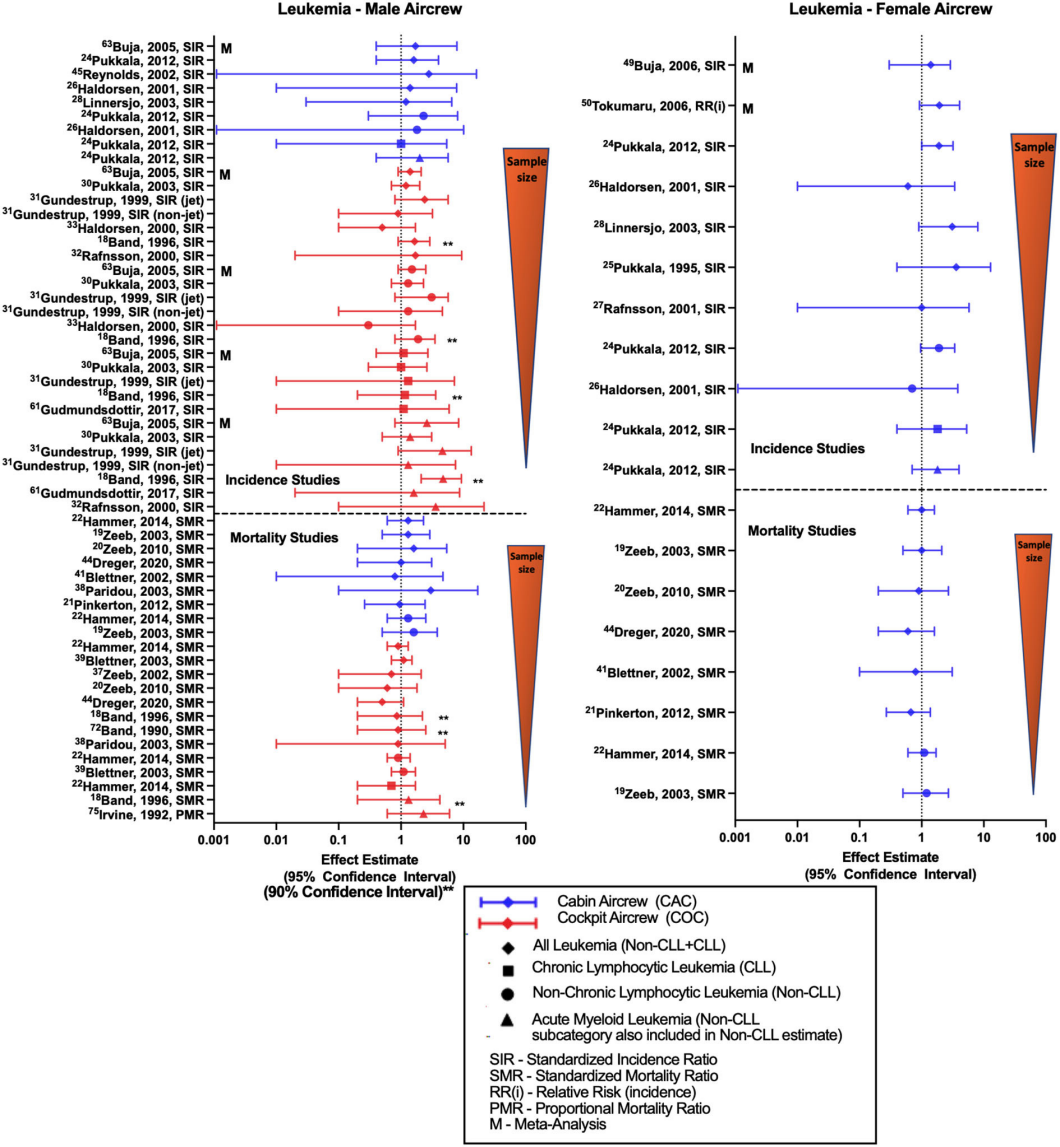
Forest plot for studies evaluating leukemia incidence or mortality ratios among male **(left hand side graph)** and female aircrew **(right hand side graph)** in association to CIR exposure.

Studies evaluating associations between work as aircrew and risk of leukemia (Figure 6) mostly report risk estimates for either all-leukemia, chronic lymphocytic leukemia (CLL), non-chronic lymphocytic leukemia (non-CLL) or acute myeloid leukemia (AML—subcategory included within non-CLL estimates but sometimes reported separately). Retrospective cohort studies and meta-analysis comparing male FAs to the general population show overall findings of non-significant positive estimates with all-leukemia, non-CLL and AML (32, 37, 45, 52, 68). The single study evaluating CLL incidence in male FAs observed no difference in the results (52). Similarly, female FA studies generally observe near-significant positive estimates with all-cause leukemia and leukemia subcategories (28, 37, 52), however two studies did show an equivocal and negative risk estimate, respectively (32, 34). Meta-analyses of all-leukemia risk in female FAs also report non-significant positive estimates (59, 60). Male pilot investigations of all-leukemia and subcategory risk generally find non-significant positive estimates consistent with meta-analysis report (68). A recently published study observed a statistically significant association of all-leukemia in a cohort of Korean “air transportation industry” male workers as compared to government employees (SIR 1.86, 95% CI: 1.15, 2.84) and all employees (SIR 1.77, 95% CI: 1.10, 2.70), however this group included additional non-aircrew occupations such as air transportation control officers, aircraft maintenance crew and ground staff, and therefore has not been included in the figure (76). A CIR dose-response evaluation of non-CLL risk was performed in the large Nordic cohort observing a non-significant positive trend per 10 mSv increase for female FAs, suggesting a dose response pattern (52). Since leukemia, especially when categorized according to subtype, is a much less frequently occurring cancer (as compared to the previous outcomes of BC, prostate cancer, skin cancer), the number of leukemia cases included in these retrospective cohort studies are modest, ranging in pilots from a single case (33) to 15 cases in the pooled Nordic analysis (54).

All-leukemia male FA mortality analyses of the ESCAPE cohort mainly demonstrated non-significant positive estimates with employment as aircrew (19, 22, 39), while the most recent follow-up of the large German cohort observed a null estimate that differed from previous analyses (20, 49). In contrast, the female FA cohorts in these studies observed null or negative estimates with all-leukemia mortality (19–22, 49, 51). ESCAPE cohort analyses also found non-significant positive estimates of non-CLL mortality and with employment as aircrew in both male and female FA cohorts (19, 22). Male pilot all-leukemia studies report no difference in the findings except for non-significant negative estimates observed in the large German cohort (20, 48, 51). ESCAPE cohort investigations of non-CLL mortality in male pilots also reported null findings (22, 55). One dose-response study evaluating a combined male cockpit and cabin crew cohort reported a non-significant positive all-leukemia estimate while observing an association by duration of employment as aircrew (*p* = 0.046) (38), however other CIR dose-response analyses of leukemia in pilots failed to show any clear evidence of a mortality trend with cumulative CIR exposure (23, 56). Overall, the leukemia incidence literature showed a pattern of non-significant positive estimates for all-leukemia, non-CLL, and AML among aircrew which contrasted with findings for mortality studies. These findings are not explained by sample size variations, as mortality studies included more cases than the incidence studies. Variations may be indicative of differential associations according to leukemia severity or with survivorship as opposed to incidence, or due to not adjusting for leukemia-specific risk factors.

### Thyroid cancer (TC)

**Figure 7:**
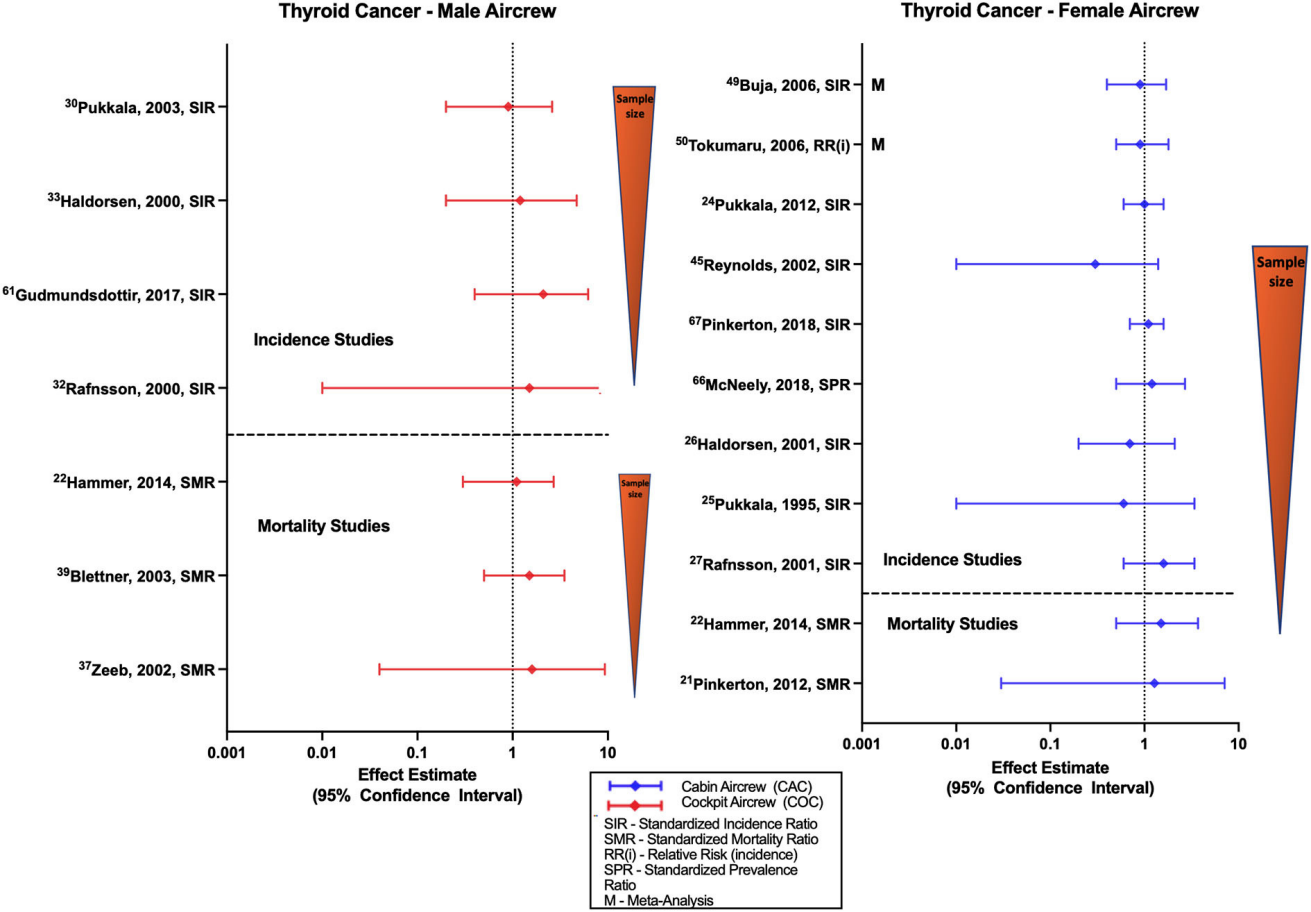
Forest plot for studies evaluating thyroid cancer incidence or mortality ratios among male **(left hand side graph)** and female aircrew **(right hand side graph)** in association to CIR exposure.

The association between TC and radiation has previously been well documented and studied in settings outside of the flight environment (77). Figure 7 summarizes studies that evaluate TC incidence and mortality risk in aircrew, which are mainly evaluations of male pilot and female FA cohorts. Studies of TC risk in male pilot cohorts generally show non-significant positive estimates (31, 33, 35) that was also reported in a mixed sex pilot meta-analysis estimate (78). Studies evaluating TC in female cabin crew are mixed, observing equivocal findings in some cohorts (46, 52, 74) and meta-analyses (59, 60, 78), as well as non-significant negative and positive estimates (34). Dose-response analysis in female cabin crew found no evidence of a risk relationship with increased cumulative CIR estimation, circadian disruption, or employment duration (46).

There are few TC mortality studies in aircrew (Figure 7). Male pilot mortality studies report non-significant positive estimates in the ESCAPE and large German cohort (48) that agree with positive estimates observed in female FA cohorts (21, 22), and with a mixed sex cohort meta-analysis of all aircrew positions (78). However, one mixed sex pilot cohort did observe a non-significant negative estimate (23). CIR dose-response analysis of male pilot mortality found no evidence of risk with increasing employment duration (55).

### Brain and central nervous system (CNS) cancer

**Figure 8:**
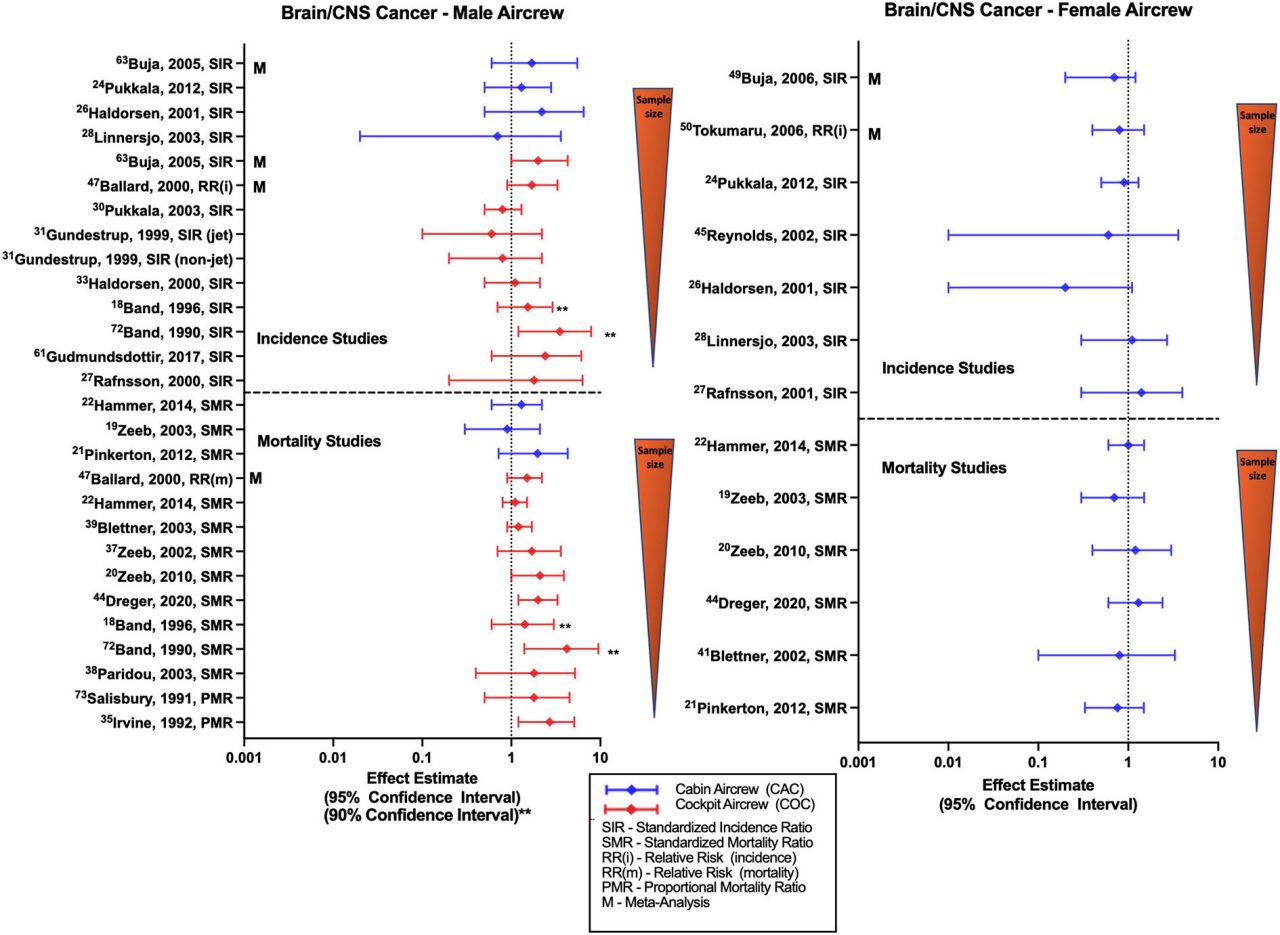
Forest plot for studies evaluating brain and CNS cancer incidence or mortality ratios among male **(left hand side graph)** and female aircrew **(right hand side graph)** in association to CIR exposure.

Chronic low doses of IR have been previously associated with brain cancer risk and persistent cognitive dysfunction (55, 79). Investigations of brain cancer and CIR exposure in aircrew generally observe non-significant varying trends and typically utilize an outcome category of “brain/CNS cancer” without further detail of cancer subtypes (Figure 8). Male cabin crew brain cancer studies consisting of the large Nordic and component studies observe non-significant positive estimates (32, 52) that agree with meta-analysis report (68). Cohort studies of female cabin crew are mixed with the large Nordic cohort and component studies observing non-significant negative (32, 34, 52) and no difference in risk estimates for brain/CNS cancer as compared to the general population. A non-significant negative estimate was also observed in a larger U.S. female FA study and meta-analyses reports (59, 60). Studies evaluating brain/CNS cancer incidence in male pilots also lack a clear trend. Smaller cohort studies report non-significant positive risk estimates (18, 31, 33, 35) that agree with meta-analyses (68) and with an observed association in a study using 90% confidence intervals (40). However, the Nordic cohort and other largest male pilot brain/CNS cancer risk study did report non-significant negative estimates (30, 54), which was also observed in a mixed cohort of male/female pilots (27).

Few studies of brain cancer mortality in male cabin crew observed non-significant positive and negative estimates in different analyses of the ESCAPE cohort and component analyses (19, 21, 22). Female cabin crew studies were also non-significant and mixed in effect estimates with ESCAPE cohort studies observing equivocal and negative estimates (19, 22), and large German cohort analyses observing positive and negative estimates (49). Most retrospective cohort studies evaluating brain/CNS cancer mortality in male pilots report non-significant positive estimates, as observed in analyses of the ESCAPE cohort (22, 39, 55). Of note, the most recent follow-up of the large German cohort did observe an association with brain cancer mortality in male pilots (SMR 2.01, 95% CI: 1.15, 3.28) (51) that was consistent with earlier non-significant estimates from the same cohort (20, 48). Individual male pilot studies also reported non-significant positive estimates with two studies observing associations based on 90% confidence intervals and proportional mortality ratio evaluations (18, 24, 40, 41). Meta-analysis assessment also observed a non-significant positive estimate (57), as did investigations of combined female/male pilot cohorts (23, 44). Findings in the pilot/ATCO study by De Stavola et al. differed, observing a non-significant negative estimate in a mixed male/female pilot cohort but did observe a mortality rate twice as high in pilots as compared to ATCOs (26). CIR dose-response analysis in male FAs observed a mortality association with duration of employment (SMR 3.20: 95% CI: 1.04, 7.47) and a non-significant positive mortality estimate with cumulative CIR dose, however a clear exposure-response relationship was lacking across all quartiles and increased risk was also observed in the highest exposure quartiles of circadian rhythm disruption (22). CIR dose-response analysis of male pilots in the large German cohort suggest a risk relationship with increasing cumulative CIR dose (50, 51) and years of employment (20, 48) that agrees with evidence from a separate study also suggesting a dose-response relationship with cumulative CIR dose estimation (23), however consistent trends have not been reported across all dose-response analyses (55). Overall studies of brain/CNS incidence and mortality in female cabin crew fail to show a consistent trend across studies, and studies of male cabin crew demonstrate a slight non-significant positive trend as compared to the general population in the few studies that have been performed. Incidence findings in studies of male pilots also do not reach statistical significance with meta-analysis evaluations and studies using employment as CIR proxy reporting positive estimates that disagree with the negative estimates observed in studies that attempt to use more robust methods to calculate CIR exposure. However, male pilot mortality studies do demonstrate a consistent positive risk estimate trend with some significance across studies as compared to the general population, and some evidence of a CIR dose-response relationship. Specific limitations in brain/CNS investigations include lack of adjustment for risk factors such as age, race/ethnicity, other potential home, or work exposures (e.g., pesticides) and family history.

### Other cancers

The frequently cited ESCAPE and large German cohorts have also reported on mortality for diagnoses grouped as radiation-related cancers (RRC). The most recent reporting of RRC in the ESCAPE cohort by Hammer et al., defines the group as including cancers of the oral cavity, esophagus, stomach, large intestine, breast, bladder/urinary tract, thyroid gland, and leukemia (excluding CLL), and observed a decreased association in male pilots (SMR 0.73, 95% CI: 0.62, 0.85), and null findings for both female and male FAs as compared to the general population (22). The most recent follow-up to the large German cohort by Dreger et al. defined RRC using the group of diagnoses defined in the ESCAPE cohort in addition to liver, pancreas, bone, non-melanoma, ovary, and CNS/brain cancer. Consistent with the findings in the ESCAPE cohort, the large German cohort noted a decreased association in male pilots (SMR 0.66, 95% CI: 0.51, 0.84), an equivocal finding for female FAs, and a non-significant negative estimate in male FAs (51). CIR dose-response analysis did yield an association of RRC in male pilots with the cumulative dose interval of 15–25 mSv (RR 2.76, 95% CI: 1.37, 6.03), however no relationships were seen within other dose intervals and therefore statistical significance was not observed across the trend test (51). The diagnosis of lung cancer was not included in the RRC group definitions due to the strong link to smoking. Some cohort studies also evaluate the risk of the individual cancer outcomes that were grouped into RRC as well as other outcomes to include cancers of the larynx, lung, testis, kidney, and eye, however these outcomes are not consistently studied across the literature and including all reported risk estimates is beyond the scope of this review. Furthermore, these outcome estimates are reflected in the all-cancer evaluations that generally report lower risk in aircrew as compared to the general population, as discussed earlier in this section.

### CIR exposure and cancer risk in military aircrew

Due to different flight environments, health effects of CIR exposure associated with military flight are assessed separately from commercial airline travel in this review. Military flight has additional factors such as unique acceleration forces, weapon/radar equipment and life support systems that may introduce different health risks, and military aircraft are flown differently depending on the specific airframe and mission requirements with respect to flight frequency, duration, and altitude. Military aviators are screened and trained differently than commercial pilots and can be subjected to more job-related stress due to contingency and combat operations. In addition to these unique considerations, military aircrew are subjected to most of the same exposures as commercial pilots and FAs (except for regular interactions with civilian passengers).

Studies that have investigated health outcomes in military aircrew are detailed in Table 2. The literature is comprised generally of retrospective U.S. cohort studies in addition to a few case-control studies, and most use occupation as aircrew for CIR exposure proxy, with a few studies stratifying by flight hours and aircraft type. Findings are mixed, both in significance and direction of results. Meta-analysis based on three military pilot studies reports association with MSC and service as a military pilot (SIR 1.43, 95% CI: 1.09, 1.87), however after a correction for socioeconomic status based on flight personnel being the “highest social class”, the finding was no longer significant (68). The meta-analysis also reported an association with NMSC (SIR 1.80, 95% CI: 1.25, 2.58) and non-significant positive estimate with brain cancer. In contrast, the meta-analysis also reported a decreased association with HL (SIR 0.51, 95% CI: 0.30, 0.89), a non-significant negative estimate with all-cause leukemia, and null finding for NHL (68). Two case-control studies reported a non-significant positive estimate (82) and an association (RR 1.51, 95% CI: 1.05, 2.18) with brain cancer in military pilots that agreed with the meta-analysis, however this trend was not observed in a recent retrospective cohort study that compared U.S. Air Force fighter pilots to matched U.S. Air Force officers (83). It is interesting to note that other than the outcome of brain cancer, the recent pilot study by Robbins et al., generally supports the meta-analysis findings to include outcomes of melanoma (after socioeconomic status correction), HL, NHL, and all-cause leukemia (68, 83). The limited studies that have evaluated prostate cancer risk in military pilots have observed non-significant positive estimates (81, 83). Cohort studies evaluating testicular cancer risk have either reported equivocal (83, 84, 87) or positive estimates (36, 86), with one small case-control study finding a significantly increased OR 1.74 (95% CI: 1.04, 2.92) (85).

**Table 2:** Cancer outcomes of CIR exposure in military aircrew populations.

**Reference** **Type of Study**	**Participant** **characteristics**	**Location of** **cohorts**	**Cases**	**Male association (95%** **confidence int)**
Buja et al. (68) meta-analysis	3 male COC studies	–	–	COC SIR: Melanoma−1.43 (1.09, 1.87) NM−1.80 (1.25, 2.58) HL−0.51 (0.30, 0.89) NHL−1.07 (0.73, 1.55) Leukemia−0.81 (0.48, 1.39) Brain−1.10 (0.47, 2.59)
Nishikawa (80) case-control	80,587 COC	United States	COC Cases: Brain−44	COC RR: Brain−1.51 (1.05, 2.18)
Rogers et al. (81) retrospective cohort	61,844 male AC	United States	AC Cases: Prostate−74	AC HR: Prostate−1.15 (0.85, 1.44)
Grayson and Lyons (82) retrospective cohort	59,940 male COC	United States	COC Cases: Melanoma−49 NM−36 HL−14 NHL−22 Leukemia−13 Brain−14 Bladder−19 Testicular−59	COC SIR: Melanoma−1.50 (1.11, 1.98) NM−1.45 (1.02, 2.01) HL−0.51 (0.29, 0.86) NHL−1.0 (0.7, 1.6) Leukemia−0.89 (0.49, 1.52) Brain−0.71 (0.39, 2.07) Bladder−2.1 (1.3, 3.3) Testicular−1.0 (0.8, 1.3)
Robbins et al. (83) retrospective cohort	4,949 COC (FP)	United States	COC Cases (FP): Melanoma−24 HL−2 NHL−5 Leukemia−3 Brain−4 Bladder−2 Testicular−19 Prostate−2 Thyroid−2	COC RR (FP): Melanoma−1.02 (0.64, 1.62) HL−0.59 (0.13, 2.74) NHL−1.15 (0.41, 3.21) Leukemia−0.84 (0.18, 3.91) Brain−0.97 (0.33, 2.84) Bladder−0.79 (0.16, 3.89) Testicular−0.92 (0.56, 1.52) Prostate−1.29 (0.31, 5.44) Thyroid−1.31 (0.30, 5.71)
Hammar et al. (36) retrospective cohort	2,808 male COC	Sweden	COC Cases: Melanoma−9 NM−17 HL−1 NHL−9 Leukemia−3 Brain−14 Bladder−7 Testicular−5	COC SIR: Melanoma−1.1 (0.5, 2.0) NM−2.1 (1.2, 3.4) HL−0.5 (0.0, 2.9) NHL−1.1 (0.5, 2.2) Leukemia−0.5 (0.1, 1.5) Brain−1.7 (0.9, 2.9) Bladder−0.5 (0.2, 1.0) Testicular−1.7 (0.6, 4.0)
Grayson and Lyons (84) case-control	127 male AC	United States	AC Cases: Brain−37	AC OR: Brain−1.2 (0.8, 2.0)
Yamane and Johnson (85) case-control	121 male COC	United States	COC Cases: Testicular−31	COC OR: Testicular−1.74 (1.04, 2.92)
Milanov et al. (86) retrospective cohort	unknown male COC	Bulgaria	AC Cases: NM−5 Bladder−4 Testicular−2	AC SIR: NM−3.1 (1.0, 7.1) Bladder−10.5 (2.9, 27.0) Testicular−1.6 (0.2, 5.8)

*COC, cockpit crew; AC, aircrew; FP, fighter pilot, NM, non-melanoma skin cancer; HL, Hodgkin's lymphoma; NHL, Non-hodgkin's Lymphoma; RR, risk ratio; HR, hazard ratio; SIR, standardized incidence ratio; OR, odds ratio.

Due to the mixed approaches and limited volume of studies, it is difficult to draw conclusions from the available literature on cancer risk in military pilots. Although there are notable differences in screening, training, exposure factors and flight profiles between male military and commercial pilots, there do exist some similarities when comparing cancer incidence outcome trends. The general association with MSC and NMSC is shared between military and commercial pilot studies as well as a non-significant positive trend in prostate cancer incidence. The reports for NHL seen in military pilots is similar to NHL risk estimates seen in commercial pilots, however the limited but increased association seen in commercial pilots with HL contrasts with the decreased HL association reported in military pilots. This disagreement was also demonstrated in all cause leukemia incidence, with the negative trends seen in male military pilots contrasting with positive trends observed in male commercial pilots. The single study that evaluated thyroid cancer risk in military pilots did agree with the non-significant positive trend observed in commercial pilot studies, and brain cancer trends in military studies generally mirror the conflicting brain cancer incidence risk estimates discussed in male commercial pilots previously. It is important to consider that many military pilots eventually find work in the civilian sector, creating overlapping cumulative exposures from various compositions of flight environment factors. Investigation into different factors of flight-related exposure in commercial aircrew have found evidence that military service is a significant exposure source, and therefore it is important to understand these associated health effects even when evaluating commercial aircrew (88).

## Limitations and challenges of linking CIR exposure to health effects

Due to the complexities of studying the health effects of chronic intermittent low dose exposure to CIR among the multitude of factors that exist in the flight environment, most epidemiologic studies share similar limitations.

### Study approach and selection

The literature on health risks associated with CIR exposure is dominated by retrospective cohort studies, which can be problematic as these rely on existing records that could include missing or incomplete information since the data was not collected for the purposes of research. Additionally in retrospective studies it can be more difficult to establish an exposure-outcome temporal relationship. Although most studies have used the corresponding geographically matched general population as a control, however the general population is likely not an appropriate comparison group due to lower rates of risk factors and comorbidities in aircrew such as tobacco use, overweight/obesity status, diabetes, hypertension, hyperlipidemia, and cardiovascular disease, and a higher socioeconomic status (4, 89). This is an example of the “healthy worker effect” and likely arises in part due to the physically and psychologically demanding work of aircrew (90). The healthy worker effect can diminish the apparent risk associated with working as flight crew. On the other hand, in comparing aircrew to the general population, there is potential for overestimating associations because of more frequent medical surveillance among aircrew leading to higher rates of detection (91). Nevertheless, the regular medical surveillance paired with occupational medical standards also applies a “healthy survivor effect” with only healthy survivors being retained in the workforce (92). The cohorts are mostly from the Northern Hemisphere and Western countries, primarily the U.S. and Europe. Due to overrepresentation of white male pilots and white female FAs, it may not be possible to generalize the findings to aircrew of different ethnicities or non-western backgrounds and regions. Many cohorts evaluated in the literature are notably young, which is expected to lower the observable numbers of cases and deaths and limit the power of these studies to detect small effect sizes. The lower representation of female pilots and male FAs across studies also create challenges in understanding significant trends.

### Exposure measurement

In addition to study design, cohorts, and control populations, exposure dosimetry and estimations also contribute limitations to epidemiological findings. Measuring CIR exposure is challenging because of the multiple particle types and energies involved (10). Conventional CIR dosimeters have historically been expensive and cumbersome, whereas personal/portable dosimeters are much less sensitive and fail to measure the full range of particles included in CIR (10, 93). Measurement studies employ dosimeters that may not be able to comprehensively record the variable particle composition and low dose levels of CIR experienced during flight, and there has been a lack of dosimeters aboard aircraft to collect exposure data on both single flights as well as individualized cumulative aircrew doses over an entire year or a career. Many studies employ a variety of methods to estimate cumulative CIR doses over the course of aircrew careers, exposure misclassification is a concern across the literature and would be expected to push studies to observe null findings. In lieu of direct dosimetry to evaluate individual CIR exposure in aircrew cohort studies, some investigations employ group-level exposure strategies which include self-reported vs. company flight logbooks (route, duration, frequency, block hours, aircraft type), employment as aircrew, employment duration and exposure matrices (job, domicile). Accurate CIR exposure estimation using flight hours requires scrutiny as historical records can report time from departing to landing gate (block hours) or total time of the actual flight, and either may not account for various ascent/descent and cruise altitude specifics. Additionally, aircrew flight records could lack information about commuter flight legs, excluding a potentially important source of exposure (94). Another consideration of historical cohorts is the evaluation of health outcomes in aircrew groups with flight exposure prior to and after initiation of the jet era when CIR exposure is expected to increase based on higher altitudes of flight, however studies evaluating this assumption have not found evidence of increased risk (19, 21, 62). An issue observed between evaluating cockpit vs. cabin crew occupational groups is that flight hours for pilots are historically well-tracked and documented by commercial airlines while FA work hours have not been similarly recorded. For studies that utilize modeling to estimate CIR exposure, there are challenges with different models and even different versions/upgrades of models being used historically across studies that account for variable estimates of CIR reported in the literature over time, and there has also been some debate over appropriate weighting factors used in estimations, specifically in the case of the predominant neutron particles that comprise CIR (95). Furthermore, not all models have available measurements or capability to estimate all flight routes or are able to account for differences occurring between airframes that could account for ascent/descent and cruising altitude parameters. In terms of dosimetry units, there are challenges with studies using effective dose measures in place of the dose equivalent, leading to accuracy concerns from the application of incorrect radiation weighting especially in the case of high-LET radiation, and introducing potential error from overestimation of carcinogenic impacts of CIR (51, 96). Another issue that was previously mentioned is that most modalities of exposure assessment via measurements or estimations do not account for SPE activity, which although rare could be extremely important in understanding health risks due to the potentially high CIR doses that can be experienced if exposed. Based on CIR dosimetry and estimation challenges combined with the reality that the retrospective cohort approach provides the most feasible method for evaluation, studies generally lack comprehensive cumulative exposure data linked to health outcome data. It is critical to note that as epidemiological studies vary widely in CIR exposure dosimetry and estimation techniques, a limitation of any review attempting to summarize trends across the literature is that explaining methodological differences between individual studies undermines the ability to draw conclusions across the literature.

### Factors and confounders

In addition to these exposure limitations, many studies in the literature have incomplete adjustment for age, tobacco and alcohol use, obesity/body mass index, lifestyle factors (behavioral and recreational activities, etc.), and cancer-specific risk factors (e.g., MSC—history of sunburns, family history, UV exposure, etc.). The factor of age presents a challenge as adverse health risks from CIR are heavily related to age with higher exposure accumulating over longer careers. As previously discussed, there are many other exposures that challenge the ability to accurately study health effects beyond the overall “flight environment” to completely understand the impact of CIR alone. Possibly the most significant of these exposures is circadian rhythm disruption, which has been recognized as a Group 2A “probable human carcinogen” (97). In addition to general circadian disruption concerns, there are lifestyle implications associated with traveling shiftwork such as fatigue, dietary habits, exercise, and mental health stressors such as relationship factors. The magnitude of other “flight environment” exposures can vary across studies based on flight characteristics specific to the cohort being evaluated but are also temporally variable, which is demonstrated by the example of tobacco exposure. Historically, aircrew were exposed to secondhand smoke from tobacco use in flight prior to the initial 1989 smoking ban on U.S. domestic flights of <6 h duration, with progression to 97% of all flights to and from the U.S. being smoke-free by 1999 (98). While this exposure does not impact future risk for newly employed aircrew and travelers, the effect of secondhand smoke exposure should be considered when discussing previous studies that evaluated exposed samples of aircrew prior to current regulations. FAs' exposure to secondhand smoke was 6–7 times that experienced by ground-based workers employed in jobs with secondhand smoke exposure, and 14 times the exposure of an average person (98).

### Outcome measurement

In evaluating health outcomes, some mortality studies have been limited by median employment of <10 years among study participants, which is incompatible with the long induction and latency periods of some cancers (65), however many outcomes do occur after the end of exposure (flying career). Reports of low-mortality cancers (breast, prostate, melanoma, NMSC, thyroid) are challenged by low numbers of deaths creating difficulty in establishing significant relationships. There are also methodological concerns arising from inconsistency in the period of CIR exposure considered. In some studies, data are recorded up to the date of diagnosis, but it is ideal to include estimates of CIR exposure only up to ~1 year prior to diagnosis. This practice avoids bias due to potential decreased exposure levels and flight activity during the symptomatic period and medical workup prior to diagnosis.

## Discussion: CIR considerations for aircrew

Overall, the epidemiological literature provides little consistent evidence directly linking CIR exposure alone to cancer. However, study results do establish increased associations of certain cancers from occupational exposure to the flight environment and even suggest association with CIR for some outcomes. Evidence is mixed when stratifying based on aircrew position and gender, which is likely due to differing gender susceptibilities and physiology, lifestyle factors, in-flight exposures (predominantly female cabin crew and male cockpit crew) and methods of flight exposure estimations (e.g., better recordkeeping for pilots). Even with these differences, melanoma and NMSC associations are observed across male and female cockpit and cabin aircrew cohorts. There is evidence of a CIR dose-response relationship for melanoma, however this is pattern has yet to be fully disentangled from the impact of circadian rhythm disruption. There is also consistent evidence of increased BC in female FAs based on incidence studies, however no CIR dose-response relationship has been observed, and in addition to the same circadian rhythm disruption concerns, there is also modification by reproductive history. Brain/CNS cancer studies provide some weak evidence of increased mortality risk in male pilots. Leukemia incidence studies yield a non-significant trend suggesting higher risk in female FAs, and a weaker trend for male FAs, but there is no evidence of a CIR dose-response relationship. On initial glance lymphoma studies demonstrate a strong association for NHL risk in male FAs, however this link is weakened by concern for misclassification errors related to increased HIV/AIDS disease as discussed previously. Thyroid and prostate cancer studies were mostly equivocal, failing to show any consistent trends, similar to the RRC analyses. The limited studies of cancer outcomes in military pilots show some consistency with commercial pilot cohort trends in MSC, NMSC and brain cancer risk.

Although work as aircrew has been recognized as an occupation having some of the highest levels of radiation exposure, aircrew exposure is expected to increase in the future. Pre-COVID era ICAO data showed that the annual worldwide total number of passengers has increased by 187% over the last 20 years, from 1.47 billion to 4.23 billion (99). Over the same period, flight durations and frequency of circumpolar routing has also steadily increased, leading to higher CIR exposure for aircrew and passengers. Commercial jet manufacturers are now producing aircraft that regularly fly over 15-h flights, and airlines continue to push for longer routes as seen in Qantas's “Project Sunrise” test flight taking 19 h and 16 min and traveling 9,900 miles between New York and Sydney (100).

Future public travel will likely expand to include supersonic and suborbital passenger flight, each having its own challenges with respect to CIR exposure. It has been estimated that for supersonic flight, the benefits of decreased CIR exposure due to shorter flight times would outweigh the increased exposure at higher altitude (101). However, this exposure reduction can be offset by the need for transpolar routing and is dependent upon the state of the solar cycle. Similar challenges exist in estimating exposure in suborbital flight that would travel near an altitude of 62.5 miles (330,000 feet) above sea level, with CIR levels similarly dependent on a variety of factors, most notable being the decreased benefit of shielding from Earth's atmosphere and magnetic field. Adding supersonic and suborbital flight profiles to commercial air travel could significantly increase aircrew and passenger CIR exposure, and potentially require updates to current exposure limit recommendations. As these technological advancements are incorporated into the transportation industry, it will be critical to gain a more complete understanding of CIR health effects that could inform practical guidelines to effectively protect aircrew and passengers.

## Author contributions

CS, ST, and IM conceived, designed, and drafting the review. MS and SS contributed to graphical presentation. ZN and EM edited the review. All authors contributed to the article and approved the submitted version.

## Funding

This review project has received support by Pilot Project Grant from Harvard-NIEHS Center for Environmental Health (P30ES000002) and funding assistance from the Department of Environmental Health, Harvard T.H. Chan School of Public Health. In addition, ST and ZN were also supported by UO1ES029520.

## Conflict of interest

The authors declare that the research was conducted in the absence of any commercial or financial relationships that could be construed as a potential conflict of interest.

## Publisher's note

All claims expressed in this article are solely those of the authors and do not necessarily represent those of their affiliated organizations, or those of the publisher, the editors and the reviewers. Any product that may be evaluated in this article, or claim that may be made by its manufacturer, is not guaranteed or endorsed by the publisher.
